# The activity of daily living (ADL) subgroups and health impairment among Chinese elderly: a latent profile analysis

**DOI:** 10.1186/s12877-020-01986-x

**Published:** 2021-01-07

**Authors:** Yangchang Zhang, Yang Xiong, Qiuhua Yu, Shisi Shen, Li Chen, Xun Lei

**Affiliations:** 1 Research Center for Medicine and Social Development, Collaborative Innovation Center of Social Risks Governance in Health, School of Public Health and Management, Chongqing Medical University, Chongqing, China; 2 Andrology lab / Department of urology, the West China Hospital, Sichuan University, Chengdu, China; 3 Department of Nursing, Chongqing Medical University, Chongqing, China; 4 The First School of Clinical Medicine, Chongqing Medical University, Chongqing, China; 5 The School of Public Health, Peking University, Beijing, China

**Keywords:** ADL, IADL, Disability, Impairment

## Abstract

**Background:**

Disability in aged people became one of the major challenges in China due to the acceleration of population aging. Nevertheless, there were limited methods to appropriately discriminate the degree of combined basic activity of daily living (BADL) and instrumental activity of daily living (IADL). The present study explored an empirical typology of the activity of daily living (ADL) and its association with health status among the elderly in China.

**Methods:**

Data throughout the Chinese Longitudinal Healthy Longevity Survey (CLHLS) was retrieved and Latent profile analysis (LPA) was conducted to identify the subgroups of ADL for included elderly subjects. Multinomial regression was performed to detect the effect of identified characteristics with ADL subgroups, and the restricted cubic spine was drawn to show the changes in the relationship between age-specific ADL disability and BMI.

**Results:**

The overall participants (*n*=8108) were divided into three ADL classes by LPA - ‘no BADL limitation-no IADL limitation’ (Class one, *n*=6062, 75%), ‘no BADL limitation- IADL impairment’ (Class two, *n*=1526, 19%), and ‘BADL impairment- IADL impairment’ (Class three, *n*=520, 6%). Compared with the participants in Class one, the oldest-old, living without spouse, lacking of exercise, short in social activities, having experience of falls, having comorbidity of diabetes, heart disease, stroke, decreased cognitive function, depression symptom were highly associated with Class two and Class three. Additionally, malnutrition and asthma were associated with combined BADL/IADL impairment (Class three), while illiteracy was only associated with IADL impairment (Class two). Furthermore, a statistically significant U-shape association was detected between age and BADL/IADL disability (Class three vs. Class two) as well as BMI and BADL/IADL disability (Class three vs. Class one). The elderly aged 80–90 with IADL impairment were less likely to evolve into combined BADL/IADL impairment, and the elderly who were underweight or obese may have higher risk of combined BADL/IADL impairment.

**Conclusion:**

A novel functional assessment was explored based on LPA, by which elderly people could be classified into three distinct classes of combined BADL/IADL. The predictors identified with particular IADL/BADL classes could draw early attention to the onset of functional disability and enlighten targeted interventions to address consequent problems of aged people.

## Background

Aging of population, giving rise to the prevalence of chronic diseases, financial burden and labor shortage, emerging as public health concerns, has caused a considerable concern and become a great challenge to the public health in China [1]. Life expectancy at birth of Chinese has increased from 71.8 to 74.5 years among male and 76.9 to 79.9 years among female. However, it was unoptimistic that the healthy life expectancy of Chinese was documented from 64.5 to 66.6 years for males and 67.5 to 69.7 years for females during2013to 2017 [2]. World Health Organization (WHO) reported that the healthy life expectancy would be more significant than the average life expectancy as the healthy life expectancy has counted in the growth trend of the average life expectancy and chronic disease burden [3]. Meanwhile, the independent physical and cognitive ability were considered as key components of geriatric healthy life expectancy to fulfill the successful aging.

Functional ability is the capacity of the individual to live and participate in social activities depending on his own intention and preference with significant meaning [4]. An investigation on senior people in China indicated that the overall functional disability rate was up to 41.0%, with age-specific rates as 6.9, 23.6 and 42.7% for respondents aged 65–79, 80–89 and 90–99, respectively [5]. The functional ability of the elderly was usually measured with the basic ADL (BADL) and instrumental ADL (IADL). The BADL was one of the most commonly used criteria, covering basic self-care ability like bathing, dressing, eating and indoor activities [6]. In order to assess the ADL of the elderly in modern society, Lawton et al. designed the IADL scale, which represented individual’s adaption to surrounding environment, such as making calls, shopping, cooking and doing housework [7].

Given BADL/IADL has been widely demonstrated to be associated with functional ability, identifying the specific profile of BADL/IADL was an important premise to understand and discriminate the functional heterogeneity among elder people [8]. Having said that, previous studies showed that discriminating functional limitation in older adults was still confusing and obscure, with roughly extensive categories or unreasonable merge of evaluating items [9–12]. Misclassification of functional limitation might be partially attributed to inappropriate definition of cut-points for BADL/IADL in some specific context.

Latent profile analysis (LPA) is an individual-centered algorithm, which can confirming the internal association with indiscrete manifest variables and classify individuals into common profiles [13, 14]. Unobserved heterogeneity in BADL/IADL will be examined and identified by LPA in which case an optimized multidimensional BADL/IADL will be constructed rather than conventional ways. Meanwhile, previous studies indicated that BADL/IADL was associated with socio-demographics, physical/mental conditions and health-related actions [15–21], which need to be adjusted when exploring BADL/IADL risk among the elderly.

The present study was aiming to (1) explore BADL/IADL subgroups among the elderly by LPA and identify the difference therein, and (2) detect the association between functional limitation subgroups and characteristics of personal life and health status that help explain the heterogeneity of BADL/IADL.

## Methods

### Data source and participants

The data set used in the present study was retrieved from the Chinese Longitudinal Healthy Longevity Survey (CLHLS), which was carried out by Chinese Center for Disease Control and Prevention and directed by the Center for Healthy Aging and Development Studies at Peking University and Duke University. The CLHLS so far has launched eight waves of survey in 1998, (baseline)2000, 2002, 2005, 2008, 2011, 2014 and 2018, respectively. The overall sample was recruited across 23 provinces covering approximately 85% of China’s total population, and about half of the cities/counties in each province were selected as primary survey units. In that case, the CLHLS survey was deemed as the first largest longitudinal survey regarding the elderly in developing countries [22]. Participants in CLHLS were recruited by a targeted random sampling process, by which investigators firstly recruit an eligible centenarian interviewee in sampled city/county, and then matched a nonagenarian, aoctogenarian and three elders aged 65–79 nearby in the same street, village or town. The age and sex participants aged 65–99 were randomly predefined to ensure the comparability with the centenarians who were randomly coded [23]. The survey content of CLHLS participants included personal mental and physical health, lifestyle, family structure relationship and health care. CLHLS database was recognized to be of high quality because it had a robust results of reliability/validity testing, little missing data and a high response rate [24]. More information of CLHLS was detailed at: http://www.icpsr.umich.edu/icpsrweb/NACDA/studies/36179.

In order to reflect the latest classification of BADL/IADL for the elderly people in China, CLHLS participants aged 65 years old and above in the wave of the survey in 2018 were included in our analyses, while those with missing data, dementia, or elder than 105 were excluded. Accordingly, 8108 elderly individuals were selected in the present study.

### Assessment of BADL/IADL

BADL were measured with the following six subscales -*(1) Bathing; (2) Dressing; (3) Toileting; (4) Indoor moving; (5) Continence of defecation; (6) Eating.* Each item was scored from 1 to 3 (1 score representing complete independence; 2 scores representing partially dependence; 3 scores representing complete dependence). The more scores the respondents obtained, the higher BADL dependence they would be. IADL were rated with eight questions- *(1) Can you visit your neighbors by yourself? (2) Can you go shopping by yourself? (3) Can you cook a meal by yourself when necessary? (4) Can you wash clothes by yourself when necessary? (5) Can you walk a kilometer at a time by yourself? (6) Can you lift a weight of 5 kg, such as a heavy bag of groceries? (7) Can you continuously squat and stand up three times? (8) Can you take public transportation by yourself?* Item were rated on a three-point scale ranging from 1(Complete independence) to 3 (complete dependence). The more scores respondents obtained in B- and I- ADL assessment, the higher functional dependence they would have and need more external care/support from the family members or nursing staff. Previous evidence also showed that BADL and IADL could be independent indicators to effectively predicate functional disability among the elderly [25–27]. In addition, Spector et al. argued that a multidimensional structured BADL measurement could be more targeted to older people in need of nursing, and it would be feasible and practical to combine IADL into BADL measurement [28]. The CLHLS sample in 2018 showed a good internal consistency in BADL/IADL with the Cronbach’s ɑ of 0.818, and participants were defined as functional impairment when the “complete dependence” were identified with at least one item in the BADL or IADL scales.

### Coding of basic characteristics and health indicators

Basic characteristics were converted into categorical variables and digitally coded, including age (aged 65–79=0, aged 80–105=1), sex (female=0,male=1), marital status (living without spouse=0,living with spouse=1), education background (uneducated=0, educated =1), having physical exercises recently (no=0,yes=1), having social activities recently (no=0,yes=1), place of residence (urban=0, rural=1). Health variables, referring to the physical indicators (BMI and comorbidity status) and mental indicators (cognitive function and depression symptoms), were collected via self-reports and objective measurement. Chinese version of the Mini Mental State Examination (MMSE) was used to evaluate the global cognitive function. MMSE has four dimensions of cognitive orientation, calculation, recall and language capacity,with a total of 24 items scoring from 0 to 30, and the higher scores indicate a higher level of dependence for the respondents [21]. The elderly who obtained 24 scores and above were defined as “normal cognitive function”, while those scored less than 24 were evaluated to be “cognitive impairment” [29]. The 10-item Center for Epidemiologic Studies Short Depression Scale (CES-D) was adopted to measure the depression symptom, scoring from 0 to 30 with a cutoff point of 10, to distinguish normal and depressive groups [30]. BMI was calculated as weight (kg) /height (m^2^). Weight status was categorized into four types, namely, underweight (BMI < 18.5 kg/m^2^), normal weight (18.5 kg/m^2^ ≤ BMI < 24 kg/m^2^), overweight (24 kg/m^2^ ≤ BMI < 28 kg/m^2^), and obese (≥28 kg/m^2^). Comorbidities, such as hypertension, diabetes, heart disease, stroke, asthma and cancer, were logged through self-reports, as well as experience of falls [31].

### Data analysis

Descriptive analysis and statistical inference were conducted using Stata 16.0, and LPA were fitted using Mplus 7.4. The continuous variables were described using mean±standard deviation (SD) and converted into the categorical variable, and then all the categorical variables were presented using number and proportion (%). LPA was adopted to identify the potential class by BADL/IADL scores, and participants were divided into the class with an estimated LPA proportion via the homogeneity over the questionnaire responses. Each latent class was gradually allowed entering in the LPA model at a time after being tested and fitted with the previous class. The procedure of model fitting and algorithm iteration ended up when the *k* latent class was involved and the comparison with the *k-1* prior latent class was completed. Maximum likelihood estimation (MLE) was adopted to run the algorithm iteration at two stages - (1) set a starting value as zero to estimate and achieve maximum value, and (2) repeatedly estimated maximum value based on the value in the last step until the final one met the initially set aggregation standard [32].

The best latent classes was finalized when the test values of Akailke’s Information Criterion (AIC), Bayesian Information Criterion (BIC) and sample size-adjusted BIC (aBIC) reached a relative minimum [33–35]. Bootstrap Likelihood Ratio Test (BLRT) and Lo-Mendell-Rubin (LMR) were performed to compare differential distribution of log likelihood ratio between nested models, and statistical significance (*P*<0.05) implied the *k*-class model was better than the *k*-1 model [36, 37]. Entropy, varying between 0 and 1,indicated a more accurate classification when the value was getting close to 1. Lubke and Muthén pointed that entropy below 0.60 meant that 20% of individual were misclassified, while above 0.80 meant that 90% of individuals were precisely classified [36]. Therefore, functional ability of the elderly was classified into three subgroup based on fitted indices, namely, Class one (‘no BADL limitation-no IADL limitation’, *n*=6062), Class two (‘no BADL limitation-IADL impairment’, *n*=1526), and Class three (‘BADL impairment-IADL impairment’, *n*=520).

Univariate analyses of categorical variables were processed with Chi-square test and Fisher’s exact test. Multinomial logistic regression was performed to test the association between every latent class detected by LPA and any other variables with the reference of Class one. Restricted cubic spines with three knots at the 10th, 50th, 90th percentiles were used to flexibly fit the age-BADL/IADL association and BMI-BADL/IADL association with the odds ratio (OR) and 95% confidence interval (CI).

## Results

### Basic characteristics

A total of 8108 participants were recruited in our study. As shown in Table 1, the average age of the participants was 82.46±11.01and 53.03% (*n*=4300) of participants were female. Participants living in urban areas, being educated and living with spouse accounted for 58.76% (*n*=4764), 57.84% (*n*=4690) and 47.13% (*n*=3821), respectively. There were 36.36% (*n*=2948) of participants taking physical exercises, but only 16.56% (*n*=1343) of participants had social activities recently. The measure of BMI showed that 51.60% (*n*=4184) of subjects were normal, followed by overweight (25.44%, *n*=2063), obesity (8.72%, *n*=707) and underweight (14.23%, *n*=1154). Participants with the comorbidities of hypertension, diabetes, heart disease, stroke, asthma and cancer were respectively 44.63% (*n*=3619), 11.10% (*n*=900), 17.96% (*n*=1456), 10.80% (*n*=876) and 1.54% (*n*=125), and 21.46% (*n*=1740) of the subjects reported to have fallen down. MMSE test showed that 78.48% (*n*=6363) of the subjects were declined in cognition, and CES-D test showed that 26.63% (*n*=2159) of subjects had depression symptoms. In addition, the average of combined BADL/IADL was 18.9±6.6.

**Table 1:** Sample characteristics

Variables	Mean±***SD/N(%)***
**Sex**	
Male	3808 (46.97%)
Female	4300 (53.03%)
**Age (year)**	82.46±11.01
65–79	3496 (43.12%)
80–105	4612 (56.88%)
**Place of residence**	
Urban	4764 (58.76%)
Rural	3344 (41.24%)
**Education**	
Uneducated	3418 (42.16%)
Educated	4690 (57.84%)
**Marital status**	
Living without spouse	4287 (52.87%)
Living with spouse	3821 (47.13%)
**Physical exercise (recently)**	
No	5160 (63.64%)
Yes	2948 (36.36%)
**Social activity (recently)**	
No	6765 (83.44%)
Yes	1343 (16.56%)
**BMI**	22.58±3.65
underweight	1154 (14.23%)
normal	4184 (51.60%)
overweight	2063 (25.44%)
obese	707 (8.72%)
**Experience of falls**	
No	6368 (78.54%)
Yes	1740 (21.46%)
**Hypertension**	
No	4489 (55.37%)
Yes	3619 (44.63%)
**Diabetes**	
No	7208 (88.90%)
Yes	900 (11.10%)
**Heart disease**	
No	6652 (82.04%)
Yes	1456 (17.96%)
**Stroke**	
No	7232 (89.20%)
Yes	876 (10.80%)
**Asthma**	
No	7324 (90.33%)
Yes	784 (9.67%)
**Cancer**	
No	7983 (98.46%)
Yes	125 (1.54%)
**MMSE**	
Total score	25.9±5.5
Normal	6363 (78.48%)
Cognitive decline	1745 (21.52%)
**Depression**	
Total score	7.5±4.2
Normal	5949 (73.37%)
Having depression symptoms	2159 (26.63%)
**BADL**	6.6±1.7
**BADL disability**	943 (11.63%)
**IADL**	12.3±5.4
**IADL disability**	3186 (39.29%)
**BADL/IADL**	18.9±6.6
**BADL/IADL disability**	3245 (40.02%)

### Latent classification of BADL/IADL

**Fig. 1:**
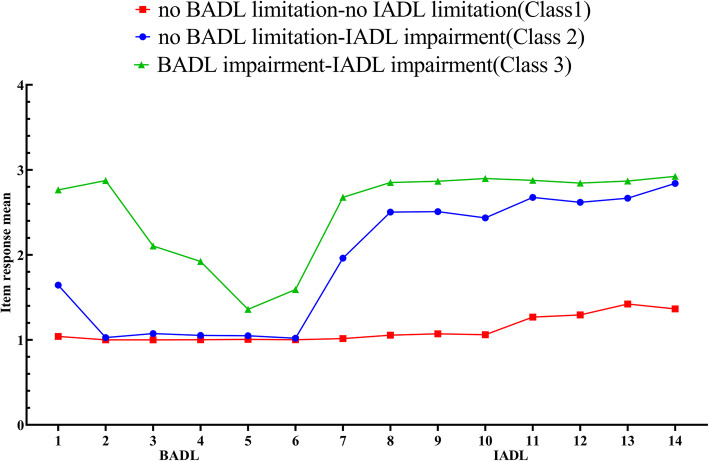
Distribution of the mean BADL and IADL scores for each of the items by subgroup. *Note.* BADL: basic activities of daily living; IADL: instrumental activities of daily living

Table 2 showed the analyses of BADL/IADL profiles for the participants. AIC, BIC and aBIC, testing the goodness of fit, were decreasing continuously when classes were gradually taken in the model. Entropy, ranging between 0.97 and 0.98, showed an optimal fitting with five models. BLRT were highly significant for 2-, 3-, 4-class model, and LMR indicated that the posterior model (3- and 4-class) were better than the previous model. In summary, 2-class model was firstly excluded because of inferior model fitting and higher AIC and BIC. LMR of 5-class model was not better than the 4-class model (*P*=0.556), and the size of two clusters in 4-class model were only 4 and 3%, therefore, 5-class LPA model was subsequently excluded. 4-class model was similar in cluster distribution with 3-class model, but the 3-class model was more concise. Eventually, 3-class model was considered to be the best according to the parsimonious principle. Figure 1 showed the pattern of the 3-class solution of BADL/IADL.

**Table 2:** Fit indices of latent class analysis on ADL subtypes

Model	K	AIC	BIC	aBIC	Entropy	LMR	BLRT	Class Probability
**1**	**28**	**182,235.96**	**182,431.98**	**182,343.02**	**–**	**–**	**–**	**1**
**2**	**43**	**120,223.44**	**120,524.47**	**120,387.83**	**0.97**	**0.023**	**<0.001**	**0.78/0.22**
**3**	**58**	**85,654.45**	**86,060.49**	**85,876.17**	**0.98**	**<0.001**	**<0.001**	**0.19/0.75/0.06**
**4**	**73**	**73,010.91**	**73,521.96**	**73,289.98**	**0.99**	**<0.001**	**<0.001**	**0.18/0.75/0.04/0.03**
**5**	**98**	**60,971.16**	**61,587.22**	**61,307.57**	**0.98**	**<0.556**	**<0.001**	**0.18/0.62/0.13/0.04/0.02**

*K* freedom of model, *AIC* Akaike Information Criterion, *BIC* Bayesian Information Criterion, *aBIC* adjusted Bayesian Information Criterion, *LMR* Lo-Mendell-Rubin likelihoodratio test; *BLRT* Bootstraplikelihood ratio test

Table 3 presented the estimation of item response mean of BADL/IADL subtypes for each class. Class one, with the largest size (*n*=6062, 75%), had a low item response mean in both BADL and IADL (BADL mean=1.00, ranging from 1.001 to 1.039; IADL mean=1.184, ranging from 1.015 to 1.401), and it was labeled as ‘no BADL limitation-no IADL limitation’. Class two (*n*=1526, 19%) had a low item response mean in BADL (mean=1.131, ranging from 1.016 to 1.634) but a high item response mean in IADL (mean=2.502,ranging from 1.941 to 2.832), and it was therefore labeled as ‘no BADL limitation-IADL impairment’. Class three (*n*=520, 6%) was relatively high in item response mean in both BADL and IADL (BADL mean=2.041, ranging from 1.292 to 2.861; IADL mean=2.822, ranging from 2.608 to 2.914), which was labeled as ‘BADL impairment-IADL impairment’.

**Table 3:** Item response mean and standard errors in latent profile analysis of ADL

Item	Class One		Class Two		Class Three		Total sample Mean (SD)	
	Mean	SE	Mean	SE	Mean	SE	Mean	SD
	***n***=6062 (75%)		***n***=1526 (19%)		***n***=520 (6%)		***n***=8108 (100%)	
**BADL**	**1.001**		**1.131**		**2.041**			
1	**1.039**	**0.003**	**1.634**	**0.025**	**2.736**	**0.025**	**1.260**	**0.634**
2	**1.001**	**0.001**	**1.025**	**0.005**	**2.861**	**0.017**	**1.126**	**0.470**
3	**1.001**	**0.001**	**1.062**	**0.007**	**2.001**	**0.030**	**1.077**	**0.319**
4	**1.002**	**0.001**	**1.043**	**0.006**	**1.826**	**0.031**	**1.063**	**0.278**
5	**1.006**	**0.001**	**1.041**	**0.006**	**1.292**	**0.024**	**1.030**	**0.189**
6	**1.002**	**0.001**	**1.016**	**0.003**	**1.522**	**0.031**	**1.038**	**0.225**
**IADL**	**1.184**		**2.502**		**2.822**			
1	**1.015**	**0.002**	**1.941**	**0.028**	**2.608**	**0.035**	**1.291**	**0.668**
2	**1.049**	**0.005**	**2.460**	**0.026**	**2.820**	**0.023**	**1.427**	**0.764**
3	**1.066**	**0.005**	**2.474**	**0.028**	**2.842**	**0.021**	**1.444**	**0.782**
4	**1.052**	**0.004**	**2.409**	**0.031**	**2.878**	**0.018**	**1.424**	**0.770**
5	**1.249**	**0.008**	**2.667**	**0.018**	**2.852**	**0.020**	**1.618**	**0.829**
6	**1.278**	**0.008**	**2.612**	**0.019**	**2.820**	**0.022**	**1.628**	**0.842**
7	**1.401**	**0.009**	**2.666**	**0.016**	**2.846**	**0.019**	**1.731**	**0.842**
8	**1.339**	**0.011**	**2.832**	**0.013**	**2.914**	**0.016**	**1.720**	**0.891**

Class one: ‘no BADL limitation-no IADL limitation’; Class two:‘no BADL limitation- IADL impairment’; Class three: ‘BADL impairment- IADL impairment’

### Subgroup analyses

As shown in Table 4, the majority of subjects in Class two were females (*n*=951, 62.32%) while half of subjects in Class one were males (*n*=3021, 49.84%). About 93.45% of Class two were oldest-old people, as twice as it was in Class one (*n*=2714, 44.77%). Urban residents were dominated in Class three (*n*=358, 68.85%). The illiterate took the largest proportion in Class two (*n*=974, 63.83%) while the subjects living with a spouse was least in this class (*n*=300, 19.66%). The subjects having physical exercises (*n*=52, 10.00%) and social activities (*n*=26, 5.00%) took the smallest part in Class three. The elderly in Class three were high in underweight (*n*=133, 25.58%) and experiencing falls (*n*=170, 32.69%), and subjects in Class one were high in overweight and obesity (*n*=2289, 37.76%). Furthermore, the proportion of subjects having heart disease (24.42%), stroke (22.50%), asthma (16.15%) and cancers (1.92%) were largest in Class three, as well as the proportion of cognitive decline (66.54%) and depression symptoms (39.81%). Subjects with hypertension (45.65%) and diabetes (11.55%) accounted for the most in Class one. There was statistically significant difference among subjects in Class one, two and three in all the included variables except for having cancer.

**Table 4:** Difference among ADL types with included variables

Variables	Class one	Class two	Class three	***P*** value
**Sex**				<0.001
Male	3021 (49.84%)	575 (37.68%)	212 (40.77%)	
Female	3041 (50.16%)	951 (62.32%)	308 (59.23%)	
**Age (year)**				<0.001
65–79	3348 (55.23%)	100 (6.55%)	48 (9.23%)	
80–105	2714 (44.77%)	1426 (93.45%)	472 (90.77%)	
**The place of residence**				<0.001
Urban	3472 (57.27%)	934 (61.21%)	358 (68.85%)	
Rural	2590 (42.73%)	592 (38.79%)	162 (31.15%)	
**Education**				<0.001
Illiterate	2146 (35.40%)	974 (63.83%)	298 (57.31%)	
Educated	3916 (64.60%)	552 (36.17%)	222 (42.69%)	
**Marital status**				<0.001
Living without spouse	2644 (43.62%)	1226 (80.34%)	417 (80.19%)	
Living with spouse	3418 (56.38%)	300 (19.66%)	103 (19.81%)	
**Physical exercise (recently)**				<0.001
No	3443 (56.80%)	1249 (81.85%)	468 (90.00%)	
Yes	2619 (43.20%)	277 (18.15%)	52 (10.00%)	
**Social activity (recently)**				<0.001
No	4836 (79.78%)	1435 (94.04%)	494 (95.00%)	
Yes	1226 (20.22%)	91 (5.96%)	26 (5.00%)	
**BMI**				<0.001
underweight	690 (11.38%)	331 (21.69%)	133 (25.58%)	
normal	3083 (50.86%)	837 (54.85%)	264 (50.77%)	
overweight	1725 (28.46%)	258 (16.91%)	80 (15.38%)	
obese	564 (9.30%)	100 (6.55%)	43 (8.27%)	
**Experience of falls**				<0.001
No	4942 (81.52%)	1076 (70.51%)	350 (67.31%)	
Yes	1120 (18.48%)	450 (29.49%)	**170 (32.69%)**	
**Hypertension**				<0.001
No	3295 (54.35%)	901 (59.04%)	293 (56.35%)	
Yes	**2767 (45.65%)**	625 (40.96%)	227 (43.65%)	
**Diabetes**				0.037
No	5362 (88.45%)	1385 (90.76%)	461 (88.65%)	
Yes	**700 (11.55%)**	141 (9.24%)	59 (11.35%)	
**Heart disease**				<0.001
No	5012 (82.68%)	1247 (81.72%)	393 (75.58%)	
Yes	1050 (17.32%)	279 (18.28%)	**127 (24.42%)**	
**Stroke**				<0.001
No	5482 (90.43%)	1347 (88.27%)	403 (77.50%)	
Yes	580 (9.57%)	179 (11.73%)	**117 (22.50%)**	
**Asthma**				<0.001
No	5533 (91.27%)	1355 (88.79%)	436 (83.85%)	
Yes	529 (8.73%)	171 (11.21%)	**84 (16.15%)**	
**Cancer**^**a**^				0.646
No	5968 (98.45%)	1505 (98.62%)	510 (98.08%)	
Yes	94 (1.55%)	21 (1.38%)	**10 (1.92%)**	
**MMSE**				<0.001
Normal	5407 (89.19%)	782 (51.25%)	174 (33.46%)	
Cognitive decline	655 (10.81%)	744 (48.75%)	**346 (66.54%)**	
**Depression**				<0.001
Normal	4639 (76.53%)	997 (65.33%)	313 (60.19%)	
Having depression symptoms	1423 (23.47%)	529 (34.67%)	**207 (39.81%)**	

Class one: ‘no BADL limitation-no IADL limitation’; Class two: ‘no BADL limitation- IADL impairment’; Class three: ‘BADL impairment- IADL impairment’. ^a^Fisher’s exact test

### Multinomial logistic regression

The Multinomial logistic regression was performed to calculate the OR of being a member of Class two or Class three versus Class one (Table 5). Logistic regression model showed that the elderly living in the rural area (OR=0.65, 95% CI=0.56–0.74), well-educated (OR=0.84, 95%CI=0.72–0.98), living with spouse (OR=0.48, 95%CI=0.41–0.57), having physical exercises (OR=0.39, 95%CI=0.34–0.46) and taking social activities (OR=0.45, 95%CI=0.34–0.58) were less likely to be allocated into Class two. Furthermore, subjects who were oldest-old (OR=8.26, 95%CI=6.58–10.38), suffering from diabetes (OR=1.35, 95%CI=1.06–1.72), heart disease (OR=1.27, 95% CI=1.05–1.52), stroke (OR=1.72, 95%CI=1.38–2.15), falls (OR=1.58, 95%CI=1.36–1.85), cognitive impairment (OR=3.79, 95% CI=3.26–4.40) and depression symptoms (OR=1.24, 95% CI=1.07–1.44) were more likely to be assigned into Class two.

**Table 5:** Multinomial logistic regression on ADL subgroups

Variable^a^	Class Two		Class Three	
	***OR***	***95%CI***	***OR***	***95%CI***
**Sex (Female)**	0.90	0.77–1.05	0.80	0.63–1.02
**Age (80–105)**	8.26***	6.58–10.38	4.55***	3.22–6.44
**Residence (Rural)**	0.65***	0.56–0.74	0.47***	0.37–0.59
**Education (Educated)**	0.84*	0.72–0.98	1.22	0.96–1.56
**Marital status (living with spouse)**	0.48***	0.41–0.57	0.44***	0.34–0.58
**Exercise (Yes)**	0.39***	0.34–0.46	0.19***	0.14–0.27
**Social activity (Yes)**	0.45***	0.34–0.58	0.36***	0.23–0.57
**BMI**				
**underweight**	1.07	0.89–1.27	1.34*	1.03–1.73
**overweight**	0.90	0.75–1.08	0.79	0.59–1.07
**obese**	1.16	0.89–1.52	1.57*	1.06–2.32
**Experience of falls (Yes)**	1.58***	1.36–1.85	1.71***	1.37–2.15
**Hypertension (Yes)**	0.95	0.82–1.10	0.98	0.78–1.22
**Diabetes (Yes)**	1.35*	1.06–1.72	1.51*	1.06–2.16
**Heart disease (Yes)**	1.27*	1.05–1.52	1.67***	1.27–2.18
**Stroke (Yes)**	1.72***	1.38–2.15	3.55***	2.66–4.73
**Asthma (Yes)**	1.19	0.95–1.47	1.56**	1.15–2.12
**MMSE (Cognitive decline)**	3.79***	3.26–4.40	9.57***	7.61–12.03
**Depression (Having depression symptoms)**	1.24***	1.07–1.44	1.33***	1.07–1.65

^***^P< 0.001;^**^P< 0.01;^*^P< 0.05;^a^ Reference group: Class 1.OR: Odds ratio.95%CI: 95%Confidence Interval. Class one: ‘no BADL limitation-no IADL limitation’; Class two: ‘no BADL limitation- IADL impairment’; Class three: ‘BADL impairment- IADL impairment’

Aging at 80 and above (OR=4.55, 95% CI=3.22–6.44), being underweight (OR=1.34, 95% CI=1.03–1.73), being obese (OR=1.57, 95% CI=1.06–2.32), experiencing falls (OR=1.71, 95% CI=1.37–2.15), having comorbidities of diabetes (OR=1.51, 95% CI=1.06–2.16), heart disease (OR=1.67, 95% CI=1.27–2.18), stroke (OR=3.55, 95% CI=2.66–4.73), asthma (OR=1.56, 95% CI=1.15–2.12), cognitive impairment (OR=9.57, 95% CI=7.61–12.03) and depression symptoms (OR=1.33, 95% CI=1.07–1.65) were positively associated with the allocation into Class three, while living in rural area (OR=0.47, 95% CI=0.37–0.59), living with spouse (OR=0.44, 95% CI=0.34–0.58), having physical activities (OR=0.19, 95% CI=0.14–0.27) and social activities (OR=0.36, 95% CI=0.23–0.57) were negatively associated with the allocation into Class three.

**Fig. 2:**
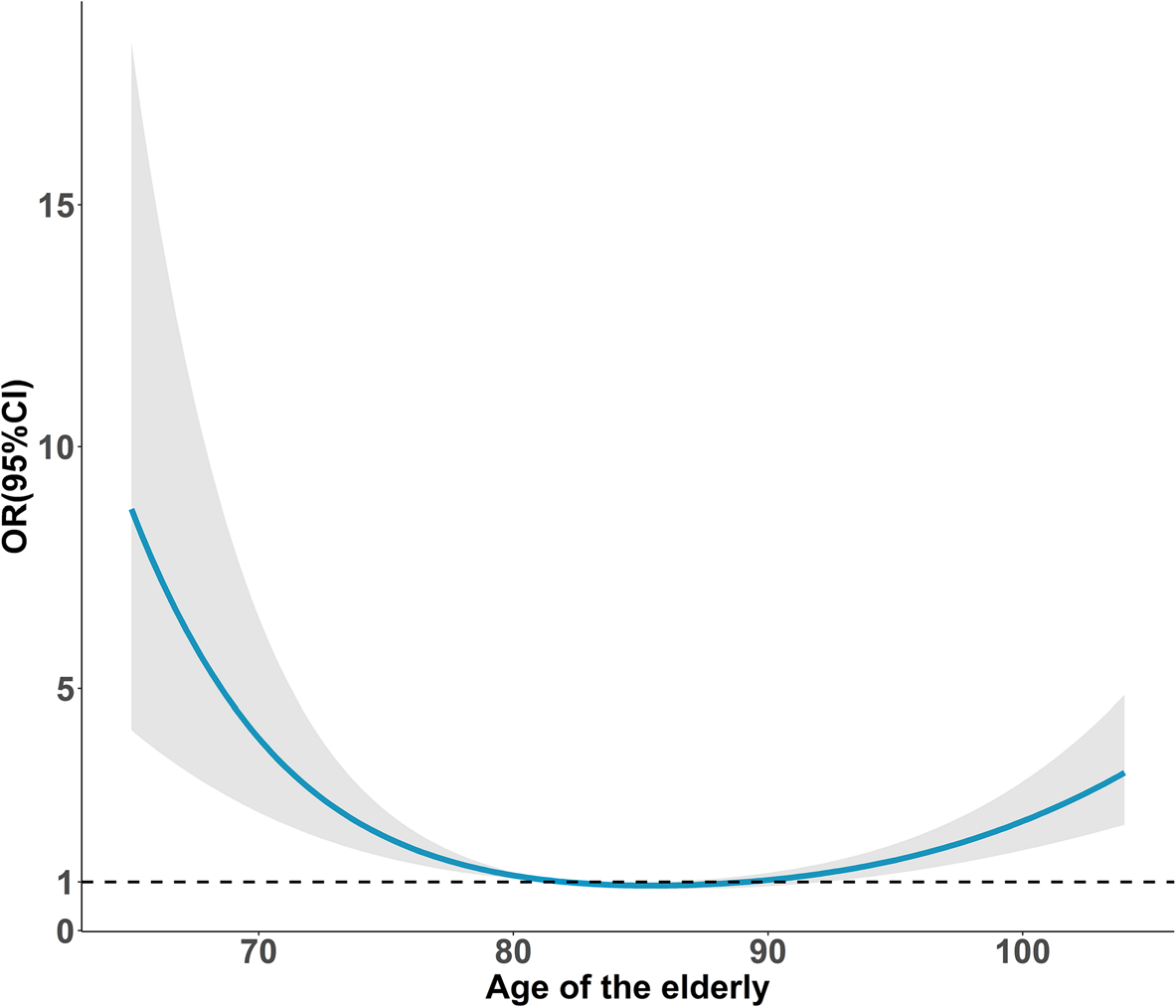
Curvilinear association between ADL disability (Class three vs. Class two) and the age among the elderly. *Note.* Shading indicates 95% CIs. The reference point is 82, after adjusting sex, fall experience, marital status, having exercise, social activities, residence place, having chronic disease, cognitive function and BMI

**Fig. 3:**
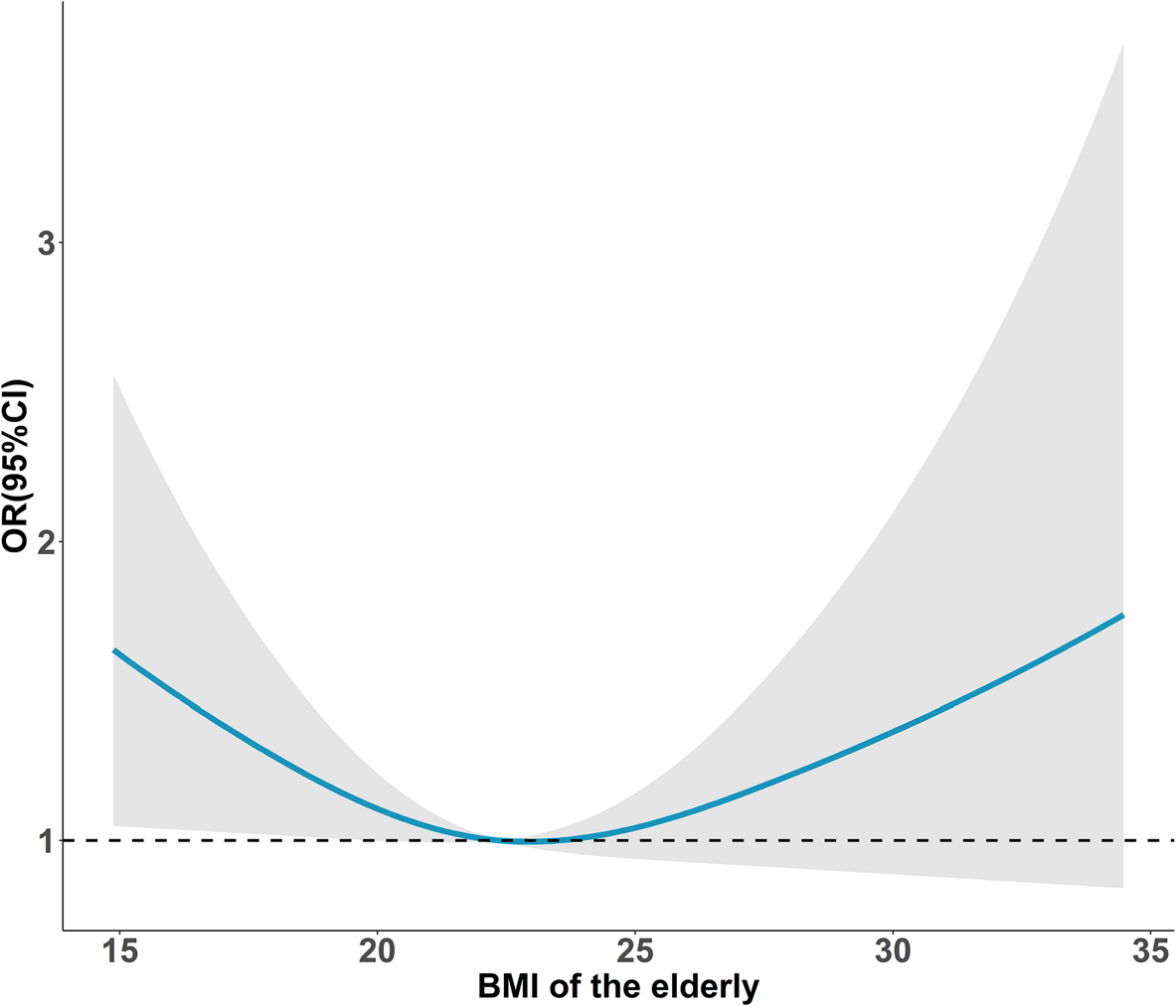
Curvilinear association between ADL disability (Class three vs. Class two) and BMI among the elderly. *Note.* Shading indicates 95% CIs. The reference point is 22, after adjusting age, sex, fall experience, marital status, having exercise, social activities, residence place, having chronic disease, cognitive function

Figure 2 showed a significant U-shape association (*P*< 0.001) between age and IADL impairment (Class three) compared to BADL impairment (Class two), and Fig. 3 showed a significant U-shape relationship (*P*< 0.001) between predicted BMI and BADL/IADL impairment (Class three vs. Class one).

## Discussion

The present study adopted LPA to explore an efficient measurement to identify the classes of BADL/IADL in the elderly in order to discriminate status of functional impairment. Three latent classes, including ‘no BADL limitation-no IADL limitation’(Class one), ‘no BADL limitation-IADL impairment’(Class two), ‘BADL impairment-IADL impairment’ (Class three), were constructed based on the item response means.

LPA clustering in our study showed that the BADL/IADL dependence (Class three) was about 6%, and IADL impairment was 19% (Class two), and 75% of participants was assigned into the almost functional independence (Class one). In contrast, the proportion of BADL impairment were lower in the studies with American population (18%) and British population (28%), while the proportion of IADL impairment was higher in the American population U.S.A (17%) and the British population (28%) [38, 39].

In line with previous evidence, age was a risk predictor for combined BADL/IADL impairment because of the age-related decline in body function and limitation in social activities [19]. The U-shape relation between age and functional impairment suggested that the elderly with IADL impairment was less likely to suffer the combined BADL/IADL impairment among aged 80–90 years. The possible reason is that the risk of having chronic diseases would also increase rapidly along with getting old, which may accelerate the ADL disability among people [40]. Meanwhile, this curve supported the view point of Hung saying that IADL impairment may increase the alertness of the elderly [39].

To our knowledge, favorable living surrounding may provide a healthier life to individuals. People living in urban area would more or less have better income, education and medical service than those in rural [41]. Zhang et al. found that inadequate access to healthcare was potential factor associated with functional limitation [42]. However, inconsistent clue was conversely detected in our study, which showed that old people in Class two and Class three were more likely to live in urban place compared with those in Class one. It is because rural residents probably take more physical activities like farming and harvesting in China, which definitely do good to the upkeep of body function.

In addition, we found that receiving education was a protective factor in Class two. Likewise, previous studies showed that the educated elderly people had better health awareness, which was significantly associated with better IADL [43, 44]. A possible reason is that the educated elderly may make better use of social support and timely seek medical care [43].

A significantly negative association between marital status and BADL/IADL impairment was basically consistent with a longitudinal study with Irish people [45], which argued that the spouse usually plays the role of caregivers particular when an elderly person has disease or functional dependence, and the ADL of the elderly would getting worse once lack of the company of the spouse [46].

Keeping moderate social activities and regular physical exercises were effective to postpone BADL/IADL disabilities [41]. According to the Activity Theory, human beings demand a sense of achievement and belonging within the social network, and the elderly were inclined to participant activities that will strengthen their social integration and adaptation [47, 48]. A cohort study in Japanese indicated that the elderly with functional disability were less engaged in community management and activities which implied that solitary status may lead to a decline of IADL function [49].

Compared with Class two, the elderly being underweight or obese were more likely to be assigned as Class three, and this association was not detected in Class one. The positive association between obese and high BADL impairment might be explained by an increasing likelihood of getting chronic diseases for the obese people which would further limit heir living function [50]. Meanwhile, underweight status was also verified to be associated with BADL impairment due to the decrement in bone mineral content and bone mineral density, which would cause a higher risk of osteoporosis and even fracture [51]. Experiencing falls were positively associated with BADL/IADL decline because the incidence of all-cause fracture will also increase among aged people who fell down [52] in line with Bahat’s conclusion, BMI was not associated with Class two, which indicated that being obese may not be a risk for IADL [53]. Partially in fact, higher BMI sometimes reflects better living standards and medical resources that may benefit IADL function.

The present study found that the diabetes, heart disease, stroke were independent risk factors in Class one and Class three. A study in Poland revealed that multimorbidity was main risk factor for BADL disability and the pains caused by the diseases may limit the body movements and result in IADL disability [54]. The elderly with diabetes often have the diabetic complication with heart, which may evolve to heart failure [55]. In addition, the elderly always have some adverse symptoms and signs, like declined activity tolerance, increased heart rate or dyspnea, which may worsen BADL/IADL impairment [56].

Depression and cognitive impairment were independently associated with BADL or IADL difficulties in Class two and Class three. Ormel et al. suggested that depression symptom and functional limitation had a mutual reinforcement among the elderly overt time [57]. The previous reports illustrated that fatigue, poor sleep quality and anorexia could reduce treatments and preventions initiative for disability, and depressive patients were undermined their IADL function [58].

Cognitive function mirrored individual’s mental status, which has interaction with physical dysfunction among age people. The reaction of external stimuli was reduced when a series of somatic diseases emerged, afterwards the cognitive damage would reversely influence the body function. Fisher et al. argued that cognitive impairment increased the risk of falling for the elderly [59]. Connolly et al. pointed out that cognitive impairment significantly decreased the function of IADL because of degenerative language and mobility [45]. Therefore, timely cognitive assessment plays an essential role in the chains between mental and physical health for the elderly.

There are several limitations that must be discussed. Firstly, the cross-sectional study limits the causality, and bi-directional relationship between healthy factors and BADL/IADL would be considered as well. Therefore, longitudinal study is in need to further explore and verify the mechanism. Secondly, recall bias was unavoidable due to the use of self-rated questionnaire. Thirdly, the subjects with missing values were excluded from analyses that will cause over- or under-estimate. And the Hawthrone effect, in which case the elderly might conceal poor performance of health and lifestyle, might disturb the risk estimate of functional disability. Finally, limited by the secondary data extraction, the hospitalization and economic data were not yet available.

## Conclusion

BADL/IADL assessment was established using the cluster LPA. Three distinct classes of combined BADL/IADL among Chinese older people were identified. The largest group was Class one (no BADL limitation-no IADL limitation) for all item of BADL/IADL scales. Stroke and cognitive impairment of the oldest-old were highly associated with BADL/IADL difficulties in Class two (no BADL limitation-IADL impairment) and Class three (BADL impairment-IADL impairment). A remarkable U-shape association was detected between age and functional impairment as well as BMI and functional impairment. Targeted interventions should be initiated in terms of the major predictors that have significant effects on the BADL/IADL classes, and well designed longitudinal studies are in need to examine the reliability and validity of the outcomes.

## Data Availability

The CLHLS questionnaires are available at https://sites.duke.edu/centerforaging/programs/chinese-longitudinal-healthy-longevity-survey-clhls/survey-documentation/questionnaires/. The full datasets used in this analysis are available from the corresponding author upon reasonable request.

## Abbreviations

BMI: Body mass index
ADL: Activity of daily living
BADL: Basic activity of daily living
IADL: Instrumental activity of daily living
CES-D: Epidemiologic Studies Short Depression Scale
MMSE: Mini-Mental State Examination
CLHLS: Chinese longitudinal healthy longevity survey
